# Unraveling the Nanomechanical and Vibrational Properties of the Mayaro Virus

**DOI:** 10.1021/acsomega.4c06749

**Published:** 2024-11-26

**Authors:** Alefe Roger Silva França, Joel Félix Silva Diniz-Filho, Clenilton Costa dos Santos, Laís Durço Coimbra, Rafael Elias Marques, Leandro R. S. Barbosa, Ralph Santos-Oliveira, Pedro Filho Noronha Souza, Luciana Magalhães Rebelo Alencar

**Affiliations:** a Physics Department, Laboratory of Biophysics and Nanosystems, Federal University of Maranhão, São Luís, MA 65085-580, Brazil; b Brazilian Biosciences National Laboratory (LNBio), Brazilian Center for Research in Energy and Materials (CNPEM), Campinas, SP 13083-970, Brazil; c Institute of Physics, University of São Paulo, São Paulo, SP 05508-090, Brazil; d Brazilian Synchrotron Light Laboratory (LNLS), Brazilian Center for Research in Energy and Materials (CNPEM), Campinas, SP 13083-100, Brazil; e Brazilian Nuclear Energy Commission, Nuclear Engineering Institute, Laboratory of Nanoradiopharmacy and Synthesis of New Radiopharmaceuticals, Rio de Janeiro, RJ 21941906, Brazil; f Laboratory of Radiopharmacy and Nanoradiopharmaceuticals, Rio de Janeiro State University, Rio de Janeiro, RJ 23070200, Brazil; g Drug Research and Development Center, Department of Physiology and Pharmacology, Federal University of Ceará, Fortaleza, CE 60356-150, Brazil

## Abstract

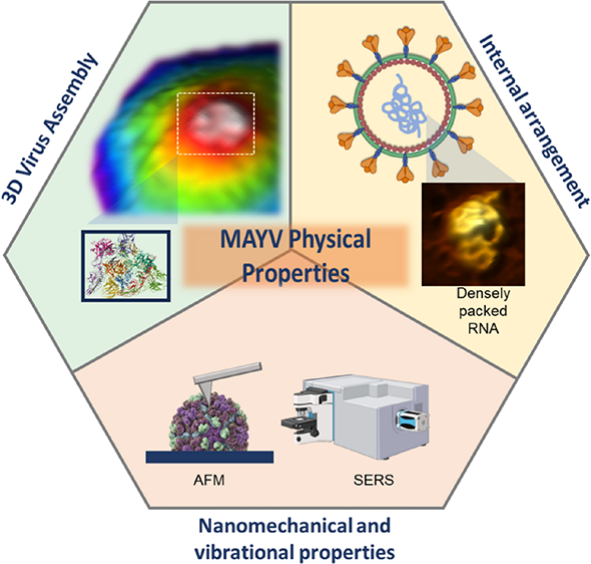

Mayaro virus (MAYV) is an emerging mosquito-borne viral pathogen whose infection results in arthritogenic disease. Despite ongoing research efforts, MAYV biology is largely unknown. Physical virology can assess MAYV nanoparticle metastability, assembly/disassembly, and polymorphism, allowing us to understand virion architecture and dynamics. Here, we employ atomic force microscopy (AFM) and surface enhancement Raman spectroscopy (SERS) to assess MAYV nanomechanical properties, including maps of adhesion force and Young’s modulus on individual viral particles. We established topographic maps of MAYV in two and three dimensions, revealing the three-dimensional arrangement and distribution of charges on viral spikes at the virus surface. Furthermore, the organization of the densely packaged RNA, which affords the viral particle exceptional mechanical resistance compared to chikungunya (CHIKV), was observed using MAYV adsorption patterns. The vibrational signature of MAYV particles differs from CHIKV, with more intense protein modes matching the distribution of E1/E2 dimers and the nucleocapsid, which are well structured and suggestive of mechanical strength.

## 1 Introduction

First described in 1954 in Trinidad and Tobago, Mayaro virus (MAYV) is an arbovirus reported in different regions of Central and South America since then.^1−3^ MAYV detection in Brazil was first reported in Amazon Forest regions and expanded to other areas.^4,5^ The identification of MAYV in samples of humans, primates, and mosquitoes in Brazil indicates that the transmission cycle is active and can potentially become an endemic arbovirus. Member of the *Alphavirus genus*, MAYV causes a nonspecific acute febrile illness with rash, retro-orbital pain, and polyarthralgia, like the arthritogenic virus Chikungunya virus (CHIKV).^6,7^ MAYV disease could evolve to incapacitating chronic clinical manifestations with joint impairment that persist for months after viremia.^8−10^

The structure of the MAYV viral particle was recently elucidated using cryoelectron microscopy (Cryo-EM)^11^ and showed a similar organization to other alphaviruses. MAYV is an enveloped icosahedral virus with ∼70 nm diameter, formed by 80 spikes in the envelope region with trimeric transmembrane glycoproteins dimers E1-E2.^11^ The genome of MAYV comprises a single-stranded RNA positive-sense surrounded by a nucleocapsid composed of copies of the Capsid protein (CP).^12^

Even with efforts to fully elucidate the characteristics and properties of MAYV, some important questions still need to be answered, such as how mechanically resistant the viral particle is, and how its molecular arrangement can influence its functions. Also, it is unknown whether these mechanical properties are shared among alphaviruses. As Cardoso-Lima et al.^13^ showed using the AFM technique, CHIKV presented an icosahedral pattern with well-defined facets, agreeing with the TEM results for the same virus^11,14^ and demonstrating the AFM’s ability to detect nanoscale patterns. However, although Ribeiro-Filho et al.^11^ suggest, through Cryo-EM technique, that MAYV must also have an icosahedral structure, the AFM results obtained here did not identify a specific geometry for these viral particles. In this scenario, Physical virology^15^ emerges to understand important properties of viral particles, such as metastability, assembly/disassembly, and polymorphism, helping to understand the dynamics of virus architecture.^13,16^

Several techniques can be employed to assess the physical properties of viral particles, such as transmission electron microscopy (TEM) and Cryo-EM, scanning electron microscopy (SEM),^17^ atomic force microscopy (AFM) and surface-enhanced Raman spectroscopy (SERS).^13,18^ Among the techniques mentioned, Atomic Force Microscopy (AFM) is the only one capable of accessing the nanomechanical properties of the isolated viral particle, from which it is possible to extract information about adhesion and Young’s Modulus (YM), the three-dimensional distribution of proteins on the viral particle and enabling the execution of nanoindentation tests. Another major advantage of the AFM technique is the possibility to obtain topographic and nanomechanical maps of viral particles in their native liquid environment,^19,20^ not requiring the sample to be fixed or treated, thus preserving all its mechanical and morphological properties.^21^

Another important point is that, since viral particles have an average diameter smaller than 100 nm, surface-enhanced Raman spectroscopy (SERS) is necessary to optimize the Raman signal on the viral particle and identify its vibrational modes.^22,22^ Christian et al.^23^ used SERS to obtain the vibrational signatures of biomolecules, including proteins and peptides from SARS-CoV-2. They were able to classify each component present in the structure of the viral particle according to their respective wavelengths and intensities, showing that SERS can inform the composition of amino acids on the viral surface. The AFM and SERS results show a strong correlation, since the nanomechanical properties of the viral particles can be associated with the interactions between proteins and lipids, in which their respective vibrational modes can be observed by SERS.^13,24^

In this work, we studied the nanomechanical and vibrational properties of MAYV particles using AFM and SERS, respectively. The two-dimensional (2D) and three-dimensional (3D) topographic maps obtained from AFM measurements made it possible to observe the ultrastructure of the viral particle. From the nanoindentation tests, it was possible to visualize rupture events associated with the breakdown of proteins that make up the viral envelope. The results obtained by SERS, through which it was possible to know the molecular signature of viral particles, corroborated what was observed by AFM since it was possible to associate each nanomechanical phenomenon observed in the sample with its composition.

## 2 Results and Discussion

**Figure 1:**
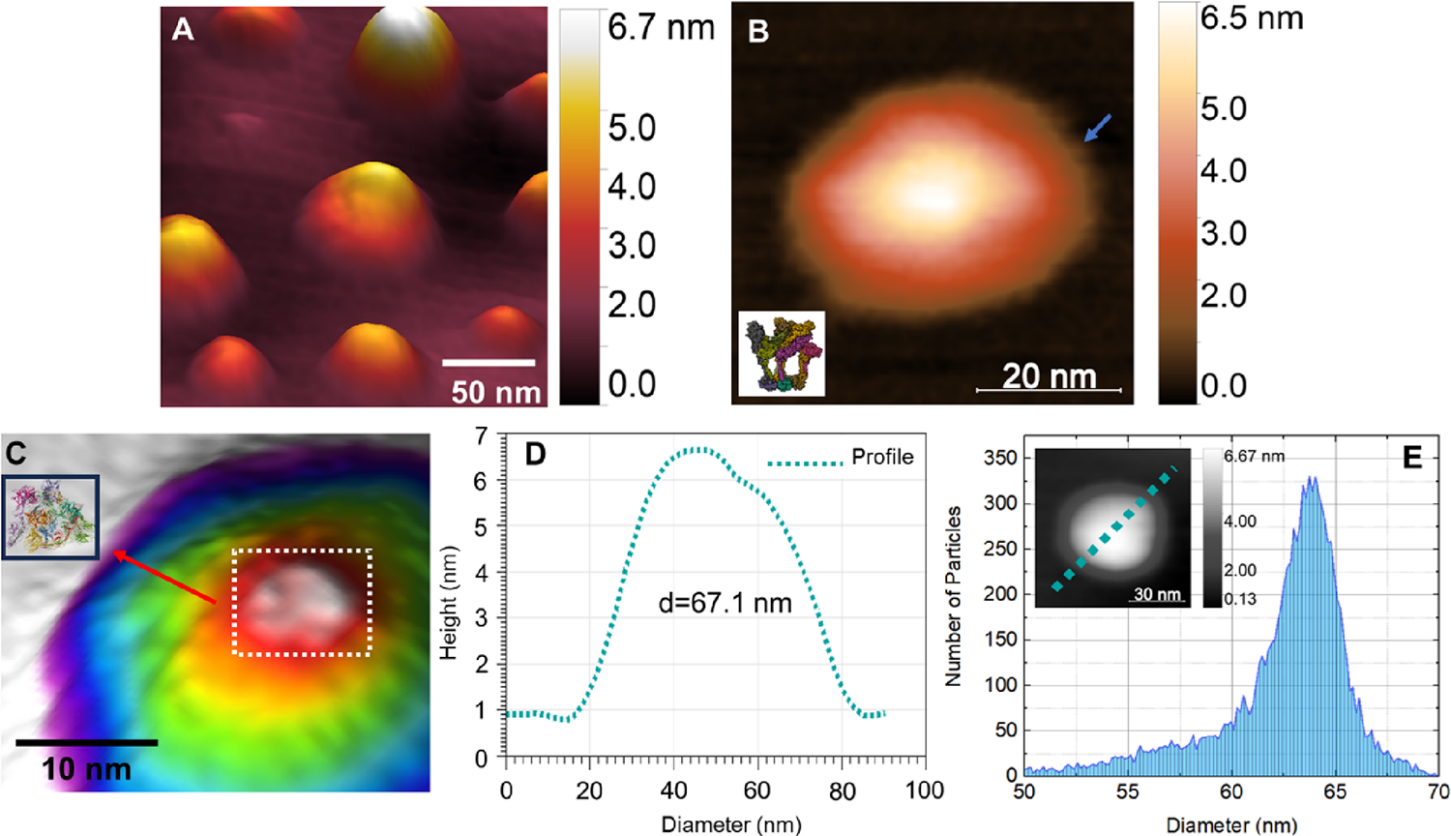
AFM image of MAYV. (A) 3D height image containing several viral particles with different adsorption patterns. (B) The viral particle shows structures suggesting spikes (blue arrow). (C) The height topographic image reveals reliefs on the surface of the viral particle related to the virus’s surface proteins. It is possible to notice a triangular structure at the top of the viral particle, resembling a MAYV spike. (D) The cross-section of the green dotted region of the detail is shown in the figure. The diameter of the particle measured in the cross-section is 67.1 nm. (E) Histogram of distribution of diameter values for MAYV viral particles. Three hundred twenty-two (322) viral particles were analyzed, and the average diameter observed was 63.8 ± 4.3 nm. The image in the inset shows the viral particle from which the cross-section shown in Figure D was taken. The insets represent the protein structures highlighted in Figures B and C, which were taken from the Protein Data Bank (PDB).^25^

Figure 1 shows topographic atomic force microscopy images of MAYV particles. Figure 1A shows a three-dimensional topographic map with different adsorption patterns of viral particles and varying diameters. The average diameter found for AFM measurements was 63.8 ± 4.3 nm (n = 322), compatible with the values observed in the literature and slightly (10%) smaller. As indicated in the work by Ribeiro-Filho et al.,^11^ MAYV presents an icosahedral geometry. Using the AFM technique, Cardoso-Lima et al.^13^ confirmed this icosahedral symmetry for CHIKV, with well-defined faces and a stable structure when adsorbed on a substrate. However, a structure that suggested well-defined icosahedral symmetry was not observed for many MAYV viral particles analyzed.

When the particles adsorb onto the substrate (mica), well-defined geometric patterns are lost. The viral envelope eventually collapses onto the nucleocapsid, forming a flattened viral particle, as shown in Figure 1B. The contrast, which represents the topography differences, allows us to visualize even a cloudier region on the edges of the viral particle, which is attributed to the extremities of the protein spikes composing the viral envelope. The blue arrow in Figure 1B highlights these structures.^11^

The three-dimensional map shown in Figure 1C reveals structures that cannot be seen in flat images. At the top of the particle, a triangular arrangement similar to an MAYV spike can be seen.^11^ The cross-section in Figure 1D is related to the dashed region in detail, with a diameter of 67.1 nm, confirming the previous observation of MAYV particle diameters. MAYV is an enveloped virus that typically has adaptable structures that preserve particle diameter even after collapse promoted by adsorption in mica.^24^

**Figure 2:**
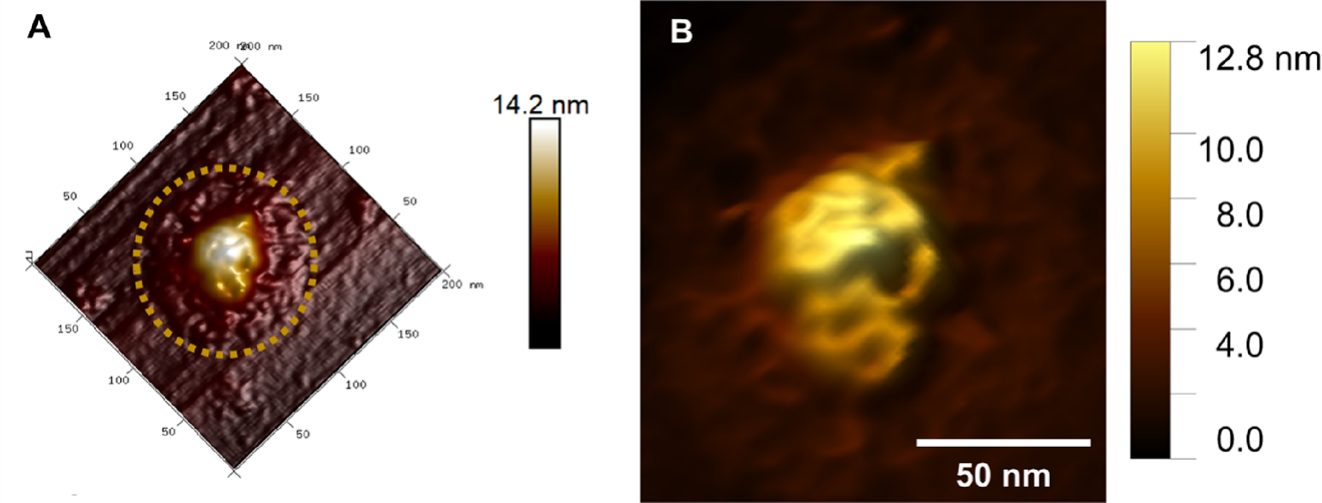
Adsorption Pattern. (A) Three-dimensional image of MAYV adhered on mica. It is possible to notice the collapsed viral envelope region, which covers the viral nucleocapsid (in light yellow). The dashed blue line delimits the collapsed particle. (B) A zoom-in image of the viral particle is shown in (A), where it is possible to visualize the MAYV nucleocapsid in greater detail, suggesting a dense RNA packaging inside the viral particle.

We show a 3D map of a MAYV particle with well-defined internal contents (Figure 2). In this contrast, we can observe the peripheral part corresponding to the protein/lipid envelope layer, as indicated by the magenta arrow (Figure 2A). This part of the viral structure is not easily visualized in two-dimensional topographic maps since viral particles are strongly adsorbed to the mica substrate. The internal nucleocapsid is less displaced by particle adsorption, generating a difference in height between the shallow edges and the elevated center of the viral particle. Thus, the internal nucleocapsid does not collapse entirely in the adsorbed viral particle. Figure 2B shows the arrangement of the internal systems in more detail. The viral RNA-C protein complexes appear to form large coils, apparently in a random distribution inside the viral particle.

**Figure 3:**
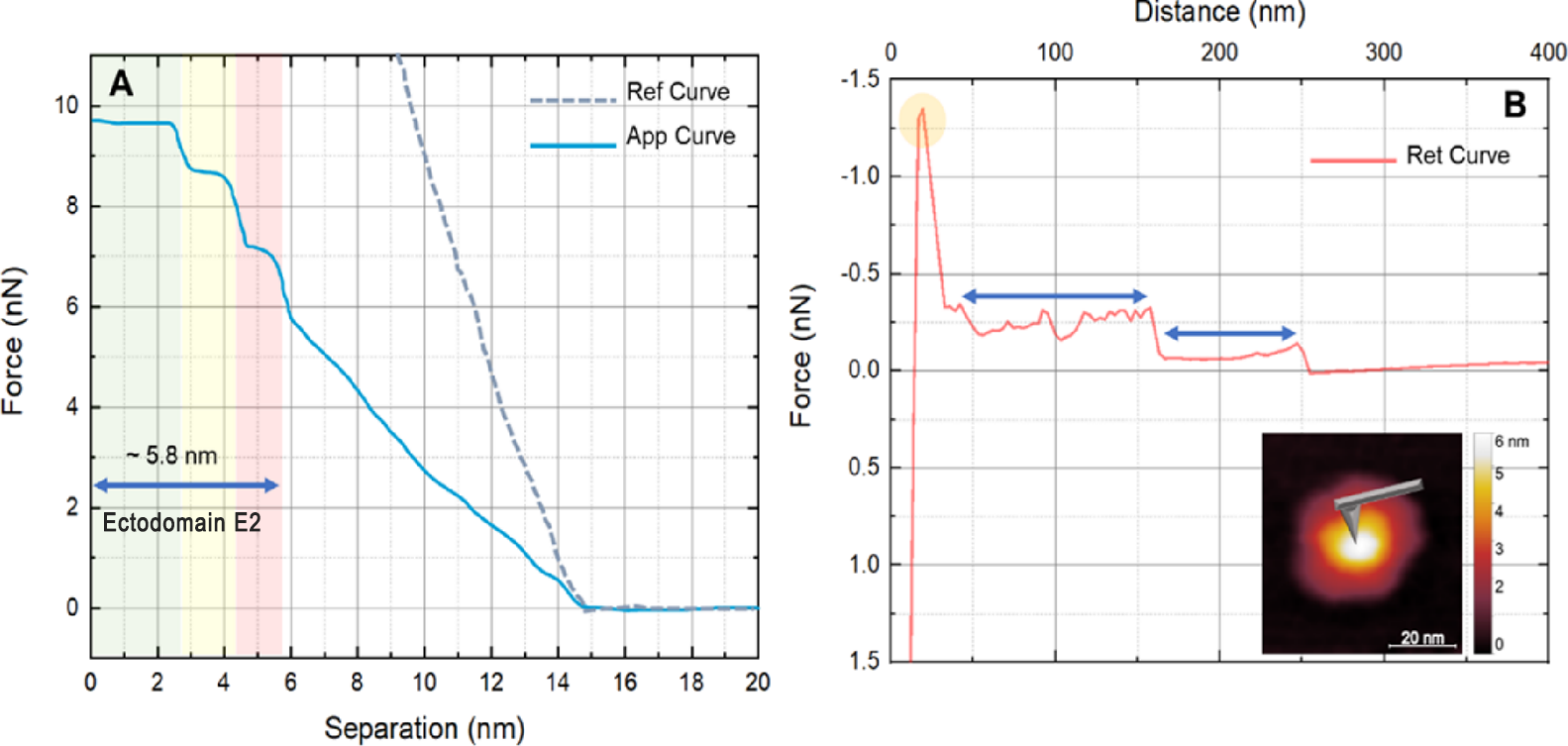
Force Curves Extracted from Nanoindentation Tests. (A) Representative approximation curve corresponding to the fourth indentation cycle on a MAYV particle. The colored bands identify three different rupture slopes with a thickness of 5.8 nm, suggestive of the layer formed by the E2 protein. The black dotted curve is the reference curve taken on the glass coverslip. (B) The retraction curve corresponds to the penetration event shown in Graph A. Three events are observed: (i) a release event (yellow circle) with a maximum adhesion force of 1.5 nN and (ii) two tethering events (blue arrows), characteristic of interaction between probes and protein layers. The same force curve behavior was observed by Lyonnais et al. for SARS-CoV-2 virions.^21^

**Figure 4:**
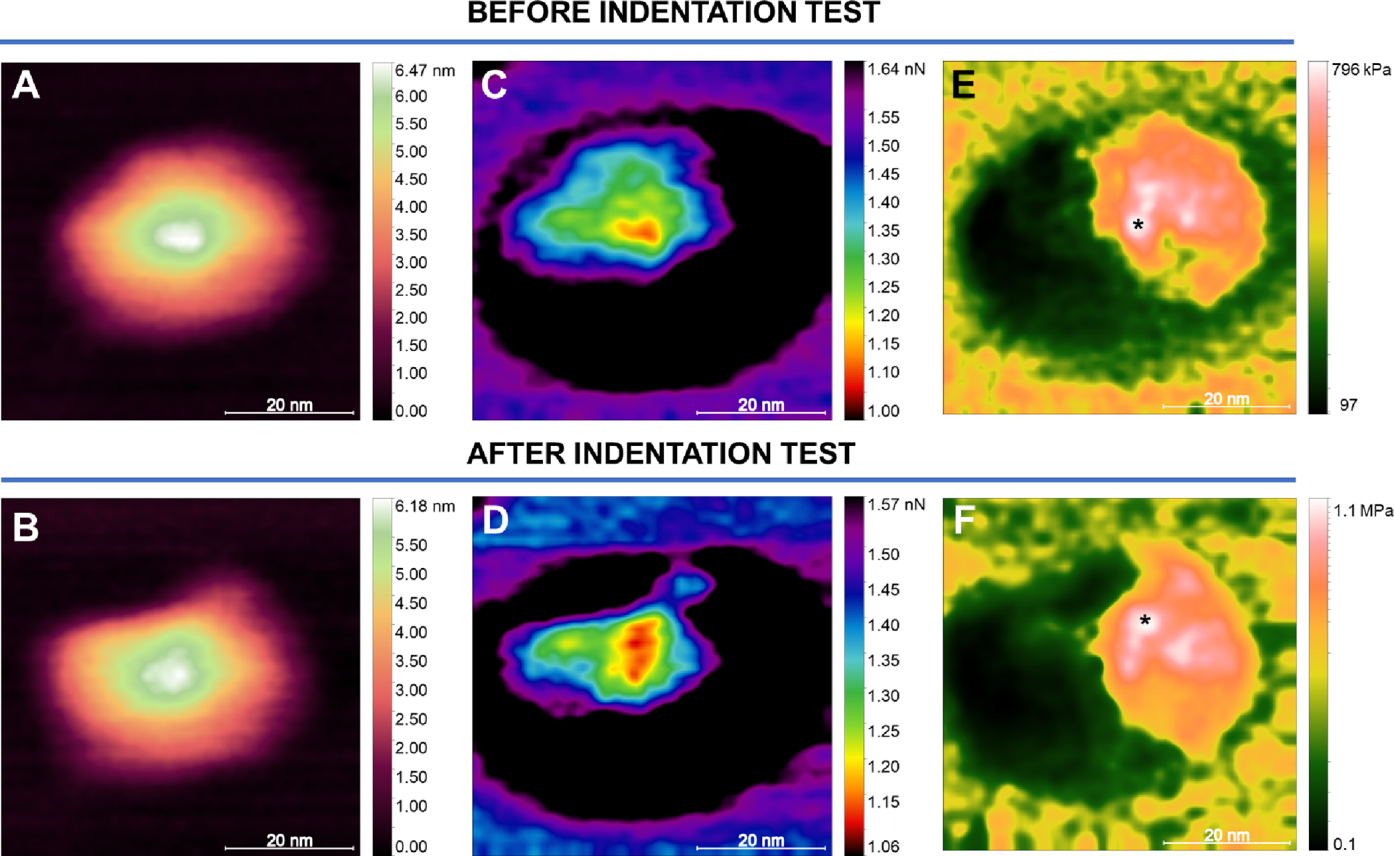
Nanomechanical Properties from an Isolated Viral Particle. Height maps of a MAYV single particle before (A) and after (B) indentation tests. Figures (C) and (D) show the distribution of adhesion forces before and after obtaining the force curves on the viral particle. Figures (E) and (F) show the Young’s Modulus maps before and after the indentation tests. The black asterisks show the internal structure’s displacement with the particle’s perturbation due to indentation.

Despite this adaptive capacity in surface adsorption processes, MAYV proved extremely resistant to mechanical damage. Figure 3 shows a representative fatigue test of MAYV using the AFM probe as the nanoindenter. Forces of up to 30 nN were applied in individual curves performed on the center of each particle, and 60 approach/retraction cycles were performed in each test. An approximation force curve over MAYV indicates the failure slopes (Figure 4A). As seen in this figure, three different breakpoints appear as the probe penetrates the layers of viral particles. These ruptures occur within a separation interval of 5.8 nm, suggesting that it may be the E2/E1 protein ectodomains, followed by the E2 and E1 transmembrane helices embedded in the envelope and the nucleocapsid core. The first structural rupture occurred with forces of 6 nN at the fourth cycle of nanoindentation. We did not observe deeper ruptures in later cycles despite the difference between the reference curve (dashed curve, gray) and the approximation curve (blue curve), indicating the MAYV particle’s deformability. Plastic deformation events were not observed, showing that MAYV is different from other arboviruses such as Zika virus (ZIKV) and CHIKV and capable of self-healing, a behavior also presented by SARS-CoV-2.^16^ Even with precise force control, only the outermost ∼5.8 nm layer can be accessed (related to the E2/E1 ectodomains).^11^

Notably, this layer can be divided mechanically into three sequential layers ruptured with forces of approximately 6, 7, and 8.5 nN, respectively (Figure 3A). This result is confirmed by the retraction curve experiments, which show three distinct release events (Figure 3B). The first release event, marked by the yellow circle, detaches from the most external layer with a maximum adhesion force of ∼1.3 nN. Next, we observed two tethering events typical of probe interaction with protein layers.^26^ Peptides attach to the AFM probe, and retraction of the tip leads to measurements of tooth-like events (blue arrows, Figure 3B), which represent the pulling forces between peptides attached to the probe and the remaining sample adsorbed to the substrate.^17^

Maps of nanomechanical properties of MAYV particles, such as adhesion and Young’s Modulus (E), were drawn to observe particle surface and structural organization before and after the mechanical fatigue tests (Figure 4). Topographic maps indicating sample height before and after are shown in Figures 4A and B, respectively, in which we noticed a change in the shape of the MAYV particle. However, we do not observe particle structure or plastic deformation disruption despite imparting local forces of up to 30 nN.

The adhesion maps before and after the curves (Figures 4C and D, respectively) also show changes in the distribution of adhesion forces. We observe the diameter of the viral particle before the fatigue test, as delimited in black and indicating the highest adhesive forces, which is larger than the image shown in Figure 3A. Furthermore, it is possible to observe a triangle-like structure on the particle’s surface with lower adhesion forces (Figures 4C, D). Cardoso-Lima et al.^13^ observed this same organization in CHIKV adhesion maps, associating this organization with the different domains of the E1 and E2 proteins. After indentations, the distribution of adhesion forces is altered (Figure 4D), demonstrating the mobility of viral membrane proteins.

Figure 4E shows Young’s modulus maps (*E*) on the MAYV particle before indentations with the AFM probe. The brightest regions reach values of ∼796 kPa. As shown in the quasi-static indentation tests, the maps obtained by PeakForce Quantitative Nanomechanics (QNM) mode^27^ show that MAYV achieves higher *E* values when compared with ZIKV,^26^ with SARS-CoV-2,^24^ and with CHIKV.^13^ Despite being extremely malleable (due to its adsorption processes), MAYV is resistant to mechanical damage, especially on top of the nucleocapsid region. After the use of force (Figure 4F), internal structures of the virus are displaced by the AFM probe, as indicated by the displaced asterisk in the map of E. As these structures become more densely packed, it is possible to observe that the maximum value of E for this map increases, reaching values above 1.1 MPa.

**Figure 5:**
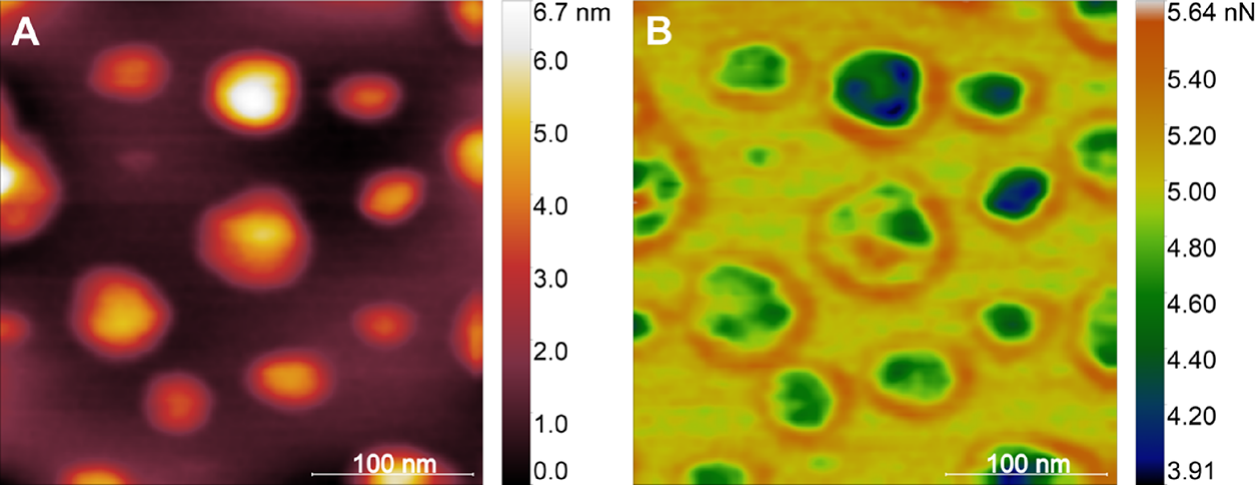
Adhesion forces. (A) Topographic map containing several viral particles adhered on mica substrate. (B) Map showing the distribution of adhesion forces on viral particles.

Adhesion maps also suggest charge distribution on the surface of the MAYV particle (Figure 5A). Regions that present lower values of adhesion forces (F_adh_), as shown in the dark regions in viral particles in Figure 5B, are likely to have negative charges since the probe is strongly electrified by friction due to the high acquisition frequencies (1kHZ) of the curves in QNM mode.^27^ We also observe that particles with larger diameters have more negatively charged regions, which may reflect an association between particle size and surface charge. Unlike other icosahedral viruses analyzed by AFM,^13,26^ which showed well-defined faces in height maps, the MAYV particles analyzed strongly adhered to the mica according to the adhesion maps.

**Figure 6:**
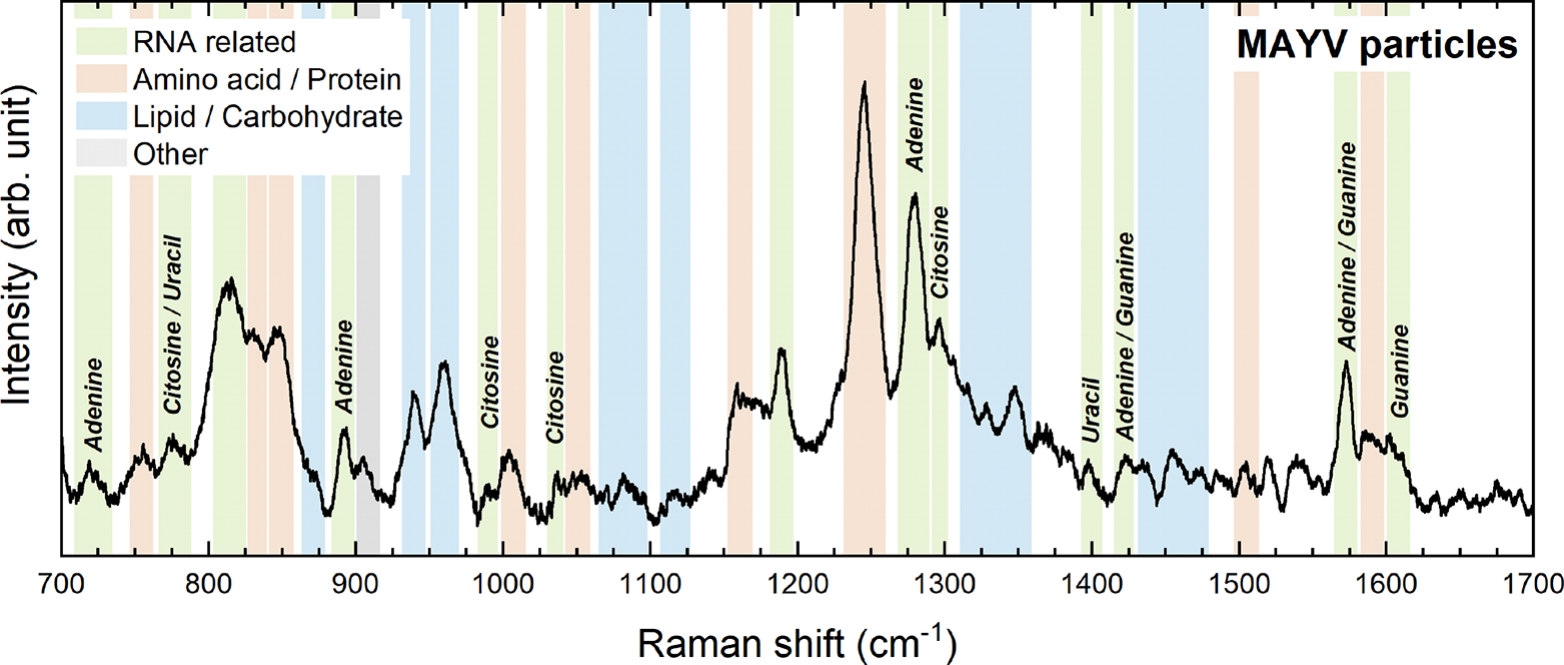
Molecular identification. Raman spectrum was obtained in a concentrated solution of viral particles adsorbed on the SERS substrate. The different colors show RNA-related modes (green), protein/amino acid-related modes, lipid/carbohydrate-related modes (blue), and others (gray).

We performed SERS experiments to identify the vibrational characteristics and molecular composition of MAYV (Figure 6). In the analyzed spectrum, we identified vibrational modes of the four bases of the viral RNA, with the modes referring to adenine presenting more intense bands when compared to the cytosine, uracil, and guanine bases. As shown by Tsika et al.,^28^ the MAYV macrodomain (MD) prefers to bind to adenine-rich RNAs, and this factor favors the interaction of MAYV with adenine-containing RNA oligonucleotides, to the detriment of uracil- or cytosine-containing RNA oligonucleotides. Also, according to Tsika et al.,^28^ the preference for adenine-rich RNAs is related to the structure and composition of the MAYV genome. Therefore, this characteristic justifies the greater intensity of the vibrational modes associated with adenine observed in the Raman spectrum of MAYV (Figure 6).

The viral RNA molecule, associated with a phosphodiester band (O–P–O), is represented by the band at 809 cm^–1^.^23^ Amino acids proline (837 cm^–1^), tyrosine (851 cm^–1^), and phenylalanine mode (1002 cm^–1^) are associated with viral proteins, together with the highest peak between 1228 and 1244 cm^–1^, attributed to amide III (CONH). Furthermore, bands corresponding to lipids (1057 cm^–1^, 1308 cm^–1^, and 1445 cm^–1^) and polysaccharides (941 cm^–1^) were identified, which reflect the composition of the viral envelope with glycosylated E1 and E2 proteins.^11^ Finally, the Amide I band was absent in the spectra, indicating metallic nanoparticles may separate protein side chains from peptide bonds in MAYV proteins.^13,29^ The exact wavelength map of each identified mode is listed in Table 1.

**Table 1:** Assignments of Each Band Are Presented on the SERS Spectrum of MAYV Particles^a^

wavenumber (cm^–1^)	RNA related	amino acid/protein	lipid/carbohydrate	other	references
722	adenine				(30)
753		tryptophan ring br.			(31)
773–782	cytosine/uracil				(31)
809	O–P–O str.				(30)
832		proline			(31)
851		tyrosine ring br.			(31,32)
870			C_1_–C_2_ str.		(30)
892	adenine				(23)
904				oleic acid	(33)
941			polysaccharides		(31,33)
960			CH_3_ def.		(30)
990	cytosine				(34)
1.002		phenylalanine			(31)
1.038	cytosine				(34)
1.043		proline			(31)
1.057–1.086			pipids		(35)
1.116			C–C str.		(30)
1.156		C–C, C–N str.			(30)
1.190	guanine				(19)
1.228–1.244		amide III			(30)
1.277	cytosine				(23)
1.296	adenine				(23)
1.308			lipids		(35)
1.328–1.348	guanine				(23)
1.397	uracil				(31)
1.424	adenine, guanine				(23)
1.445–1.456			phospholipids, lipids		(32,35)
1.503	cytosine				(34)
1.573	adenine, guanine				(23)
1.596		phenylalanine, tyrosine C=C			(31)

a Abbreviation: str. = stretching, br. = breathing, def. = deformation, bk. = backbone.

MAYV Raman spectrum compared to the CHIKV spectrum obtained by Cardoso-Lima et al.^13^ It is possible to notice similar vibrational modes, as in 809 cm^–1^, 990 cm^–1,^ and 1424 cm^–1,^ related to RNA’s structure and bases. The 851 and 1596 cm^–1^ modes related to aromatic group vibrations are present in both spectra and confirm that both viruses have aromatic amino acids composing structural proteins. This shift may be related to the high concentration of MAYV samples compared to CHIKV. Furthermore, it is important to highlight that this change may indicate structural differences between the two proteins, E1 and E2. These differences may result from variations in the amino acid sequence or other molecular characteristics specific to each virus, which aligns with what was observed in AFM results.

## 3 Conclusion and Perspectives

The results obtained in this study provide important information about the composition and structure of the MAYV viral particle. The average diameter calculated using AFM topographic maps for the viral particle was 63.8 ± 4.3 nm, a value relatively lower than that reported in the literature. Using nanoindentation tests, it was possible to evaluate the nanomechanical properties of the viral capsid, and it was found that, unlike other arboviruses such as CHIKV and ZIKV, MAYV does not exhibit the property of self-recovery. This fact can be understood by the permanent deformation of viral particles after indentation events. Another notable characteristic of the MAYV virion is its high rigidity when subjected to mechanical stress. It can withstand tensions of up to 30 nN, a very significant value compared to other viral particles. The adhesion results showed a distribution of negative charges across much of the MAYV particle surface, likely a reflection of the surface proteins.

Furthermore, SERS investigations revealed the molecular composition and vibrational properties of MAYV with adenine-rich RNA bonds. The spectral analysis also observed bands belonging to lipids, polysaccharides, and viral proteins. These findings provide valuable information about the structure and composition of MAYV, clarifying its potential interactions with host cells and immune responses. Surface proteins facilitate viral interactions with host cells.^36^ With its high sensitivity, SERS can discern even subtle changes in the conformation or distribution of these proteins when they bind to host cell receptors or when exposed to antibodies.^37^

Identifying adenine-rich RNA linkages suggests a possible role in viral replication, gene expression, and a better understanding of MAYV infection mechanisms. This information is instrumental in comprehending how the virus attaches to and enters host cells. Adenine-rich sequences in viral RNA may interact with viral or host proteins that facilitate replication.^38^ SERS can detect these sequences and their conformational changes, providing clues about how the virus replicates within the host cell. In viruses, adenine-rich regions may affect the translation of viral proteins or the regulation of viral gene expression.^39^ By characterizing these regions with SERS, it is possible to understand better how viral gene expression is controlled and how it might be targeted for therapeutic intervention. Understanding how adenine-rich RNA linkages interact with host cell machinery can reveal important aspects of the viral infection process. These regions can interact with host cell factors essential for viral entry, replication, or assembly.^40^ Similarly, SERS can identify alterations in the structure of viral particles that occur during infection or in response to host cell factors. These changes can provide valuable insights into the virus’s ability to adapt and evade immune responses.

The results discussed in this study may contribute to a better understanding of this specific biological system and help develop new drugs or antibodies to treat pathologies associated with MAYV. Establishing structural, nanomechanical, and vibrational patterns can help us understand how drugs act and modify the properties of viral particles.

## 4 Materials and Methods

### 4.1 MAYV Propagation

MAYV IQT 4235 (GenBank accession number MK070491.1) was propagated in Vero CCL81 cells cultured in DMEM (Cultilab, Brazil) supplemented with 10% v/v fetal bovine serum (FBS) and 100 U/mL Penicillin/Streptomycin (Gibco, USA) and maintained at 37 °C, 5% CO2. A cytopathic effect was observed after 24–36h inoculation with MAYV, at which point the cell culture supernatant was recovered and clarified, followed by the purification process described in Ribeiro-Filho et al.^11^

### 4.2 Atomic Force Microscopy (AFM)

AFM measurements were carried out using the methods used by Cardoso-Lima et al. for CHIKV investigation,^13^ using 10 μL of fluid containing viral particle suspensions placed on mica substrates. The virions were examined using a Multimode 8 (Bruker, Santa Barbara, CA, USA) and SNL (Bruker) probes with a notional spring constant of 0.24 N/m and a nominal tip radius of 2 nm in the peak force QNM mode. The mean diameter of MAYV was obtained by scanning a few micrometers of MAYV samples (3 maps), which could encompass many viral particles (n = 322). From this perspective, the particles are observed as small spheres projected in the *x*–*y* plane. Then, the particle size analysis function of Nanoscope Analysis 2.0 software was used to measure the average diameter, which automatically measures the diameter distribution of particles present on the 2D AFM topographic maps. Viral particle indentation tests were conducted on 11 viral particles, each subjected to 60 to 100 indentation cycles. Measurements were taken on the QNM Ramp Mode with a force set point of 30 nN and a tip velocity of 50 nm/s for the indentation analysis. The AFM data were evaluated, and the maps were created using Nanoscope Analysis and Gwyddion software.

### 4.3 Surface-Enhanced Raman Spectroscopy (SERS)

The Raman scattering measurements were performed on a micro-Raman system, model T64000 (Horiba/Jobin-Yvon), operating in the single mode, according to the procedure employed by Cardoso-Lima and co-workers.^13^ A diode laser operating at 785 nm was used as the excitation source. A neutral density filter (1%) was used to avoid laser-induced damage to the sample. The light was focused on the sample using a microscope model BX41 (Olympus), with a 100× objective lens (NA = 0.9, WD = 0.21 mm), and the Raman signal was dispersed in an 1800 gr/mm grid and detected in a liquid-nitrogen-cooled CCD. The slits of the spectrometer were adjusted to obtain a spectral resolution of 2 cm–1. A substrate with gold nanorods deposited was used as a SERS substrate. The spectrum was acquired after three acquisitions of 30 s in each dispersion band of the spectral grid.
